# Functional Role of Glutamine 28 and Arginine 39 in Double Stranded RNA Cleavage by Human Pancreatic Ribonuclease

**DOI:** 10.1371/journal.pone.0017159

**Published:** 2011-03-08

**Authors:** Md. Tabish Rehman, Punyatirtha Dey, Md. Imtaiyaz Hassan, Faizan Ahmad, Janendra K. Batra

**Affiliations:** 1 Immunochemistry Laboratory, National Institute of Immunology, New Delhi, India; 2 Centre for Interdisciplinary Research in Basic Sciences, New Delhi, India; University of Oulu, Germany

## Abstract

Human pancreatic ribonuclease (HPR), a member of RNase A superfamily, has a high activity on double stranded (ds) RNA. By virtue of this activity HPR appears to be involved in the host-defense against pathogenic viruses. To delineate the mechanism of dsRNA cleavage by HPR, we have investigated the role of glutamine 28 and arginine 39 of HPR in its activity on dsRNA. A non-basic residue glycine 38, earlier shown to be important for dsRNA cleavage by HPR was also included in the study in the context of glutamine 28 and arginine 39. Nine variants of HPR respectively containing Q28A, Q28L, R39A, G38D, Q28A/R39A, Q28L/R39A, Q28A/G38D, R39A/G38D and Q28A/G38D/R39A mutations were generated and functionally characterized. The far-UV CD-spectral analysis revealed all variants, except R39A, to have structures similar to that of HPR. The catalytic activity of all HPR variants on single stranded RNA substrate was similar to that of HPR, whereas on dsRNA, the catalytic efficiency of all single residue variants, except for the Q28L, was significantly reduced. The dsRNA cleavage activity of R39A/G38D and Q28A/G38D/R39A variants was most drastically reduced to 4% of that of HPR. The variants having reduced dsRNA cleavage activity also had reduction in their dsDNA melting activity and thermal stability. Our results indicate that in HPR both glutamine 28 and arginine 39 are important for the cleavage of dsRNA. Although these residues are not directly involved in catalysis, both arginine 39 and glutamine 28 appear to be facilitating a productive substrate-enzyme interaction during the dsRNA cleavage by HPR.

## Introduction

**Figure 1:**
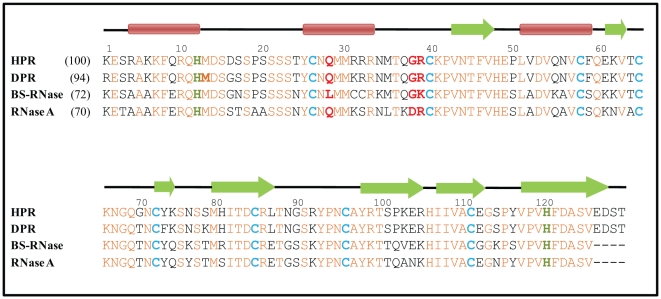
Sequence alignment of HPR with other ribonucleases. The sequences of ribonucleases were taken from protein data bank their PDB Ids being: HPR, human pancreatic ribonuclease (1DZA); BS-RNase, bovine seminal ribonuclease (1BSR); RNase A: bovine pancreatic ribonuclease (3JW1). The Swiss-Prot ID of DPR, Douc Pancreatic Ribonuclease is Q8SPN4.1. In the parenthesis is given the percent amino acid sequence similarity between different ribonucleases with respect to HPR. The secondary structures are shown at the top of sequences as, α-helices in brown filled box and β-strands in green filled arrow while the loop residues as black line. The identical residues are shown in orange and the conserved cysteine residues are highlighted in light blue. The active site residues, His12 and His119 are shaded in green while the residues under investigation, Gln28, Gly38 and Arg39 in the current study are highlighted in red.

Human pancreatic ribonuclease (HPR) is a member of an ancient superfamily of proteins, called RNase A superfamily [1]. Unlike the other members of the family, HPR displays substantial activity on double stranded (ds) RNA even under conditions in which dsRNA maintains stable secondary structure [2]. This activity is unrelated to digestion and is thought to be involved in the host-defense against pathogenic viruses [2], [3]. HPR was found to be associated with β-core preparations of human chorionic gonadotropin that showed anti-HIV replication effects [4]. Apart from HPR, bovine seminal ribonuclease (BS-RNase) and douc langur pancreatic ribonuclease (DPR), also show high dsRNA cleaving activity [5]. As compared to bovine pancreatic ribonuclease, RNase A, HPR and DPR are two logs more active on synthetic double stranded homopolymer polyA.polyU, whereas BS-RNase is one log more active [6], [7]. The members of RNase A superfamily cleave single stranded (ss) RNA by a transesterification reaction, which requires linear arrangement of the 2′oxygen atom, 5′oxygen atom and the intermediate phosphorus atom [8]. This ‘inline’ orientation of phosphodiester bond is not possible in dsRNA, which adopts helical secondary structure [9]. It was proposed that an array of positively charged residues present around the active site enables HPR and BS-RNase to destabilize the secondary structure of dsRNA thereby facilitating its cleavage [2], [9]–[11]. Figure 1 shows a multiple sequence alignment of HPR, DPR, BS-RNase and RNase A. DPR, BS-RNase and RNase A have respectively 94, 72 and 70% sequence similarity to HPR with all disulphide bonds and catalytic residues conserved (Figure 1). HPR has six additional basic residues, arginine 4, lysine 6, arginine 32, lysine 62, lysine 74 and lysine 102 around its active site, which are either absent in RNase A or replaced by non-basic residues. These unique basic residues were proposed to be responsible for the high dsRNA cleavage activity of HPR [6]. It has been shown that the mutations of arginine 4 and lysine 102 to alanine individually decrease dsRNA cleavage activity by about 2-fold, however when, glycine 38 was also mutated along with arginine 4 and lysine 102, the dsRNA cleavage was remarkably reduced [6]. Recently, we have reported that lysine 6, arginine 32, lysine 62 and lysine 74 do not play a direct role in the dsRNA cleavage activity of HPR, however lysine 6, lysine 74 and lysine 62 appear to be involved in general catalysis, structural integrity and stability and DNA helix unwinding activity of HPR [12].

A number of non-basic residues that include glutamine 28, glycine 38, proline 42, aspartic acid 83 and alanine 122 have been shown to be important for dsRNA cleavage activity of pancreatic RNases of primates [13]–[14]. We and others have shown earlier that apart from the basic residues, one non-basic residue, glycine 38 is also important for the dsRNA cleavage activity of HPR [15], [6]. RNase A lacks glycine at position 38 and instead it has an arginine at that position (Figure 1). Mutating glycine 38 to alanine caused a decrease in the activity of HPR on dsRNA [15], [6]. It was proposed that the presence of glycine at position 38 improves the flexibility of the active site cleft which in turn improves the activity of HPR and BS-RNase on dsRNA [15]. In douc langur pancreatic ribonuclease, which has similar dsRNA cleavage activity as that of HPR, glutamine 28 to leucine mutation has been shown to decrease the dsRNA cleavage activity by about 3-fold, similar to the glycine 38 to alanine mutation in HPR [13], [14]. On the other hand, this mutation in bovine background increases dsRNA cleavage activity by about 4-fold [14]. In addition, arginine 39 which is located near the active site residue lysine 41 and present in RNase A as well appears to be important for the dsRNA cleavage activity of HPR [6].

In this study we have investigated the importance of arginine 39 and glutamine 28 for the dsRNA cleavage activity of HPR. Based on the earlier studies, importance of glycine 38 has also been investigated in the context of arginine 39 and glutamine 28. Using nine variants of HPR namely Q28A, Q28L, G38D, R39A, Q28A/R39A, Q28L/R39A, R39A/G38D, Q28A/G38D and Q28A/G38D/R39A, this study demonstrates that arginine 39 is crucial for the dsRNA melting activity, and for this activity, at position 38 glycine is required. Both these residues are not directly involved in the RNA cleavage activity. At position 28, in place of glutamine, leucine is tolerated whereas alanine is detrimental for the dsRNA melting activity of HPR.

## Results

Based on studies on HPR [1], [15], BS-RNase and douc langur pancreatic ribonuclease [13], [14], Gly38, Arg39 and Gln28 residues in HPR have been proposed to be important for its dsRNA cleavage activity (Figure 1). In this study, we have probed the role of these residues in the dsRNA cleavage activity of HPR. Nine variants of HPR namely, Q28A, Q28L, G38D, R39A, Q28A/R39A, Q28L/R39A, Q28A/G38D, R39A/G38D and Q28A/G38D/R39A were prepared in which the targeted residues were mutated individually or in various combinations. Gln28 was mutated to alanine to eliminate the side chain, and to Leu to make it similar to the residue in BS-RNase (Figure 1). Similarly, Gly38 was mutated to aspartic acid to mimic the residue in RNase A (Figure 1). Arg39 was mutated to alanine to eliminate the side chain as well as the charge. In the double mutants and a triple mutant, the single mutations were incorporated in various combinations. All variants of HPR were analyzed for their ribonucleolytic activity on ds- and ss-RNA, melting activity towards a double stranded DNA and thermal stability to understand the mechanism by which these residues may be involved in the dsRNA cleavage by HPR.

### Steady-state kinetics on dsRNA and ssRNA substrates

**Figure 2:**
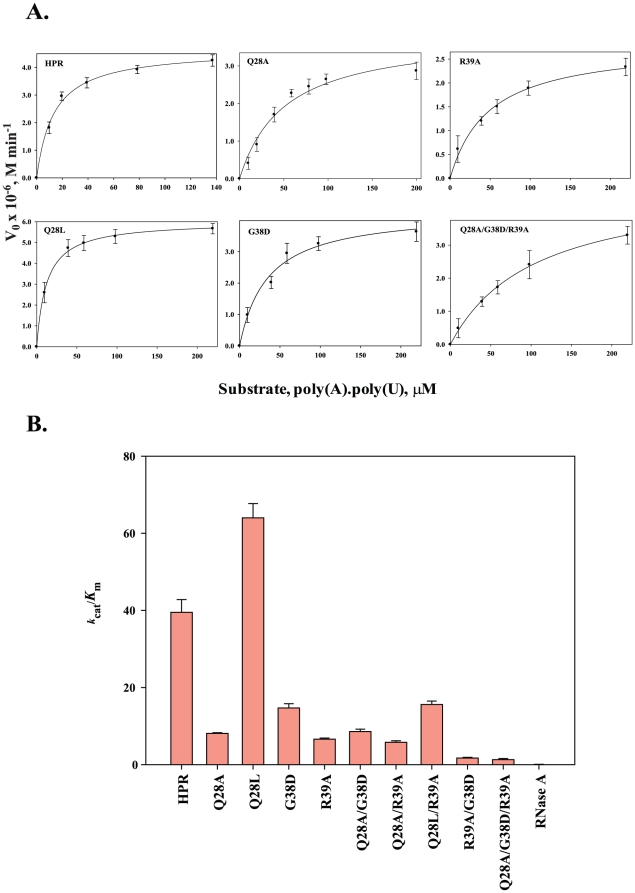
Michelis-Menten curves and catalytic efficiencies of HPR and its variants on poly(A).ploy(U). The ribonuclease activity of HPR and its variants was analysed on the double stranded RNA substrate, poly(A).poly(U) as described. A. Michelis-Menten curves; B. Catalytic efficiencies (*k*_cat_/*K*_m_).

HPR showed a 500-fold higher activity on dsRNA poy(A).poly(U) as compared to RNase A (Table 1). The *K*_m_ of HPR was about 7-fold lower and *k*_cat_ was 50-fold higher than that of RNase A for the cleavage of poy(A).poly(U) (Table 1). When Gln28 was mutated to alanine in Q28A variant there was a 5-fold reduction in the catalytic efficiency (*k*_cat_/*K*_m_) as compared to that of HPR, which was mainly due to an increased *K*_m_. Mutation of Gln28 to Leu in Q28L variant, on the other hand did not significantly affect the *K*_m_ and *k*_cat_ and accordingly there was a minor increase in the catalytic efficiency of the variant (Table 1). In R39A variant, mutation of Arg39 to Ala resulted in a 5-fold reduction in the catalytic efficiency with a 3-fold increase in *K*_m_ and 1.5-fold reduction in *k*_cat_ (Table 1). As reported earlier [15], G38D variant had about 2.5-fold reduction in its catalytic efficiency which was mainly due to a 2.5-fold increase in its *K*_m_ value (Table 1). Combining the Q28A mutation with G38D or R39A respectively in Q28A/G38D and Q28A/R39A variants did not further change the 5-fold reduced catalytic efficiency seen in the Q28A variant (Table 1). The Q28L/R39A variant behaved like the R39A variant as it had about 4-fold reduced catalytic efficiency as compared to that of the Q28L variant. Combining G38D and R39A mutations resulted in the most drastic reduction in the catalytic efficiency and the R39A/G38D variant had only 4% activity with a significant increase in its *K*_m_ and reduction in the *k*_cat_ values. A triple mutant Q28A/G38D/R39A was also almost inactive like the R39A/G38D variant (Table 1). Figure 2A shows the Michaelis-Meneten curves for HPR and few representative mutants with poly(A).poly(U) substrate. An overall comparison of catalytic efficiencies of all HPR variants with HPR on dsRNA, poly(A).poly(U) is shown in Figure 2B.

**Table 1:** Steady state kinetics of HPR and variants on poly(A).poly(U).

Protein	*K*_m_ (µM)	*k*_cat_ (min^−1^)	*k*_cat_/*K*_m_ (µM^−1^ min^−1^)	Percent *k*_cat_/*K*_m_
HPR	13.5±2.1	533.4±45.1	39.5± 3.3	100±8
Q28A	52.0±9.6	420.5±69.1	8.1±0.2	20±0.5
Q28L	11.5±0.2	736.0±37.7	64.0±3.7	162±9
G38D	34.0±3.4	498.9±23.9	14.7±1.1	37±3
R39A	48.5±5.0	320.8±18.7	6.6±0.3	17±1
Q28A/G38D	33.8±5.1	290.9±33.4	8.6±0.6	22±1
Q28A/R39A	57.2±4.7	331.5±37.9	5.8±0.4	15±2
Q28L/R39A	56.6±3.1	882.7±96.1	15.6±0.9	38±2
R39A/G38D	54.3±7.0	94.1±14.6	1.7±0.2	4±0.5
Q28A/G38D/R39A	105.9±32.6	133.9±10.0	1.3±0.3	3±1
RNase A	85.3±5.0	11.0±0.12	0.1±0.01	0.2±0.02

The kinetic parameters were obtained as described in Materials and Methods. Each experiment was done three times and the standard errors are given.

**Figure 3:**
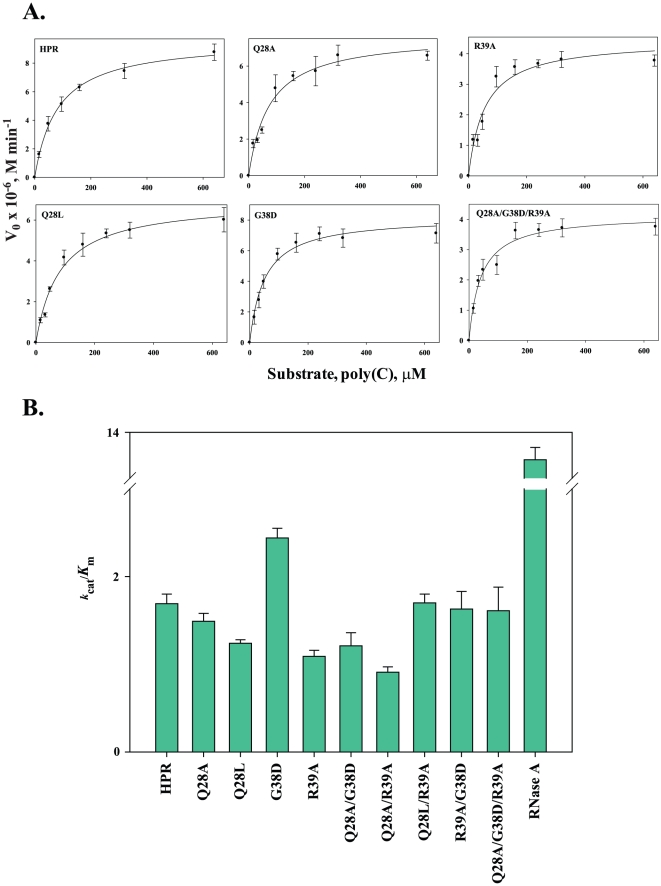
Michelis-Menten curves and catalytic efficiencies of HPR and its variants on poly(C). The ribonuclease activity of HPR and its variants was analysed on the single stranded RNA substrate, poly(C) as described. A. Michelis-Menten curves; B. Catalytic efficiencies (*k*_cat_/*K*_m_).

For ssRNA substrate, poly(C) cleavage, *K*_m_ of G38D, R39A, Q28L/R39A, R39A/G38D and Q28A/G38D/R39A was 1.5-2-fold lower than that of HPR, whereas all the other variants had similar *K*_m_. The *k*_cat_ values of all the variants were very similar to that of HPR, except for the R39A, Q28L/R39A and Q28A/G38D/R39A variants which had 2-fold decreased *k*_cat_ values (Table 2). As compared to HPR the catalytic efficiency of R39A and Q28A/R39A decreased by about 30 and 45% respectively; while for all other variants it was similar to that of HPR. The *K*_m_ of RNase A for poly(C) was very similar to that of HPR, whereas its *k*_cat_ and *k*_cat_/*K*_m_ were approximately 11- and 8-fold higher than that of HPR, respectively (Table 2). Figure 3A shows the Michaelis-Meneten curves for HPR and few representative mutants with poly(C) substrate. An overall comparison of catalytic efficiencies of all HPR variants with HPR on ssRNA poly(C) is shown in Figure 3B.

**Table 2:** Steady state kinetics of HPR and variants on poly(C).

Protein	*K*_m_ (µM)	*k*_cat_ (×10^5^, min^−1^)	*k*_cat_/*K*_m_ (×10^3^, µM^−1^ min^−1^)	Percentage *k*_cat_/*K*_m_
HPR	82.3±3.8	1.39±0.04	1.69±0.11	100±6
Q28A	73.9±5.3	1.10±0.02	1.49±0.09	88±5
Q28L	80.4±3.4	1.00±0.05	1.24±0.04	73±2
G38D	48.0±3.8	1.17±0.04	2.44±0.11	144±6
R39A	56.7±5.2	0.62±0.02	1.09±0.07	64±4
Q28A/G38D	91.0±14.5	1.10±0.02	1.21±0.15	72±9
Q28A/R39A	99.3±3.7	0.90±0.03	0.91±0.06	54±3
Q28L/R39A	43.5±3.8	0.74±0.04	1.70±0.10	101±6
R39A/G38D	60.9±10.0	0.99±0.06	1.63±0.20	96±12
Q28A/G38D/R39A	40.9±12.1	0.66±0.03	1.61±0.27	95±16
RNase A	114.2±3.5	15.3±0.73	13.4±0.27	793±16

The kinetic parameters were obtained as described in Materials and Methods. Each experiment was done three times and the standard errors are given.

### Characterization of structure and stability of HPR variants

**Figure 4:**
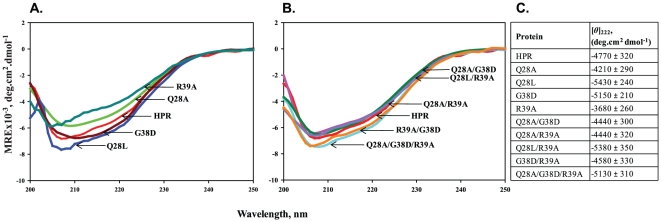
CD spectra of HPR and its variants. CD spectra were recorded in the far-UV region (200–250 nm) at pH 7.4 and 25°C. The spectra are presented as mean residue ellipticity, expressed in degrees.cm^2^.dmol^−1^. *Panel A*: CD spectra of HPR, Q28A, Q28L, G38D and R39A. *Panel B*: CD spectra of HPR, Q28A/G38D, Q28A/R39A, Q28L/R39A, R39A/G38D and Q28A/G38D/R39A. *Panel C*: [*θ*]_222_ of HPR and its variants.

The effects of the mutations on the secondary structure of the HPR and variants were studied by CD-spectral analysis in the far-UV region (Figure 4). The crystal structure of the wild type HPR is not known, however, a mutant of this protein (PM7) was crystallized, and its three dimensional structure was determined [16]. PM7 belongs to the group of α+β proteins, and it has 20% α-helix and 34% β-sheet. The CD spectral analysis revealed HPR and all its single amino acid variants (Figure 4A) and double amino acid variants (Figure 4B) to possess a compact α+β structure, with a characteristic minimum at 208 nm. The far-UV CD spectra of the variants, except for the R39A, did not show any major differences in their secondary structure as compared to HPR (Figure 4A). The R39A variant had a reduction in its helical content (Figure 4A). The far-UV CD is an excellent technique to measure conformational changes upon introduction of mutations in proteins; [*θ*]_222_ is used as a probe to measure changes in α and β structure [17]. From the far-UV CD spectra of HPR and all nine mutants, values of [*θ*]_222_ were estimated and are shown in Figure 4C. Except for the R39A mutant, which had a reduced [*θ*]_222_ value, the [*θ*]_222_ of all other mutants were not significantly different from that of the wild type protein.

**Figure 5:**
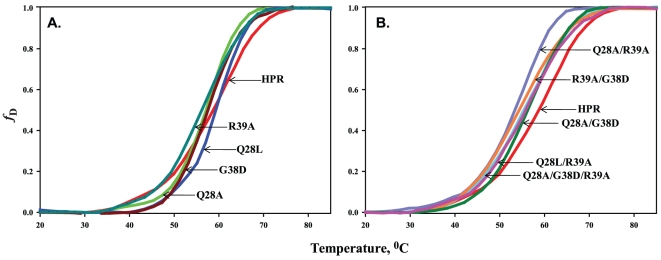
Thermal denaturation profiles of HPR and its variants. Heat induced unfolding curves of HPR and its variants are shown as plots of *f*_D_ values vs temperature. *f*_D_ is the fraction of the protein in denatured state as defined in the text. *Panel A*: denaturation profiles of HPR, Q28A, Q28L, G38D and R39A. *Panel B*: denaturation profile of HPR, Q28A/G38D, Q28A/R39A, Q28L/R39A, R39A/G38D and Q28A/G38D/R39A.

To see if the mutations affected the stability, the melting profiles of HPR and its variants were monitored by observing changes in [*θ*]_222_ in the temperature range 20–85°C. The heat-induced denaturation curves were analysed for the fraction denatured, *f*_D_ values using equation 1. Figure 5 shows plots of *f*_D_ values for HPR and its variants vs temperature. The denaturation curves for HPR and Q28L were very similar, whereas there was a shift in the denaturation curves of Q28A, G38D, R39A, Q28A/R39A, Q28L/R39A, Q28A/G38D, R39A/G38D and Q28A/G38D/R39A towards lower temperatures (Figure 5). The *T*_m_, temperature at which *f*_D_ equals 0.5, of Q28A, Q28L and G38D were very close to that of HPR (Table 3). However, R39A, Q28A/G38D, Q28A/R39A, Q28L/R39A, R39A/G38D and Q28A/G38D/R39A variants showed a remarkable decrease in their thermal stabilities (Table 3).

**Table 3:** Transition temperatures of HPR and variants.

Protein	*T*_m_, °C
HPR	58.3±0.1
Q28A	56.8±0.1
Q28L	58.9±0.1
G38D	56.5±0.4
R39A	55.9±0.1
Q28A/G38D	56.1±0.1
Q28A/R39A	54.0±0.2
Q28L/R39A	55.1±0.1
R39A/G38D	54.2±0.2
Q28A/G38D/R39A	55.7±0.1

The transition temperatures were derived from the thermal denaturation curves of HPR and its variants. Each experiment was done three times and the standard errors are given.

### Helix unwinding activity of HPR and variants

**Figure 6:**
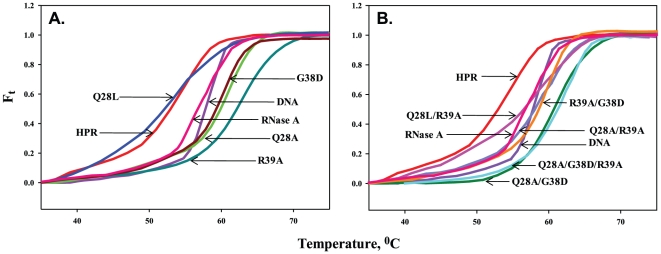
Effect of HPR and its variants on thermal transition profile of double stranded DNA poly (dA−dT).poly(dA−dT). The thermal transition profiles of DNA alone or with protein were studied spectrophotometrically at 260 nm in 10 mM MOPS buffer containing 50 mM NaCl (pH 7.5). Melted fraction of DNA (F_t_) was plotted against temperature. *Panel A*: thermal transition profile of DNA in the presence of HPR, Q28A, Q28L, G38D, R39A and RNase A. *Panel B*: thermal transition profile of DNA in the presence of HPR, Q28A/G38D, Q28A/R39A, Q28L/R39A, R39A/G38D, Q28A/G38D/R39A and RNase A.

Although no direct experimental evidence for dsRNA destabilizing activity of HPR or any other pancreatic RNase is available, dsDNA melting activity of HPR and BS-RNase is well documented, and has been extrapolated for the melting of dsRNA [5], [7]. The helix destabilizing activity of HPR and its variants was monitored on dsDNA, poly(dA-dT).poly(dA-dT). As shown in Figure 6, HPR showed a remarkable early helix destabilizing activity and this activity was not compromised by Q28L mutation. The DNA in the presence of HPR and Q28L started melting around 43°C while DNA alone started to melt around 53°C (Figure 6A). All other variants showed poor ability to melt DNA even at higher temperatures (Figure 6A, B). The melting temperatures, *T*_m_ of DNA alone, and in the presence of HPR and HPR variants are given in Table 4. The *T*_m_ of DNA alone was 57.5°C, while that with HPR was 52.6°C which was very similar to that with the Q28L variant (Table 4). However, the *T*_m_ of DNA with Q28A/R39A, R39A/G38D and Q28L/R39A variants were similar to that of DNA alone (Table 4). The helix destabilizing activity of Q28A, G38D, R39A, Q28A/G38D and Q28A/G38D/R39A was found to be much more compromised as *T*_m_ of DNA with these variants was much higher (Table 4). RNase A also was found to be poor in melting dsDNA (Table 4).

**Table 4:** Transition temperatures (*T*_m_) for the melting of DNA in the presence of HPR and variants.

Protein	*T*_m_, °C	Change in *T*_m_ compared to HPR, °C
None (DNA alone)	57.5±0.3	-
HPR	52.6±0.4	-
Q28A	59.4±0.6	6.8
Q28L	52.5±0.4	−0.1
G38D	59.7±0.5	7.1
R39A	62.4±0.3	9.8
Q28A/G38D	60.3±0.4	7.7
Q28A/R39A	58.4±0.3	5.8
Q28L/R39A	56.0±0.5	3.4
R39A/G38D	58.1±0.5	5.5
Q28A/G38D/R39A	61.7±0.6	9.1
RNase A	56.3±0.7	3.7

The transition temperatures were derived from the thermal denaturation curves of DNA in the absence and presence of HPR and its variants. Each experiment was done three times and the standard errors are given.

### In silico analysis of HPR variants

**Figure 7:**
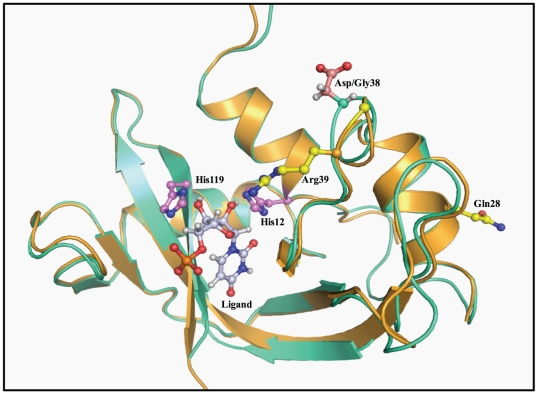
Cartoon model of HPR (golden) superimposed over RNase A (light green). The structure was drawn by taking atomic coordinates from Protein Data Bank in PyMol. PDB IDs of HPR and RNase A are 1DZA and 3JW1, respectively. All residues are shown in ball and stick model. The residues under investigation, Gln28, Gly38 and Arg39 are shown in yellow. Asp38 of RNase A is shown in red, active site residues His12 and His119 in light pink and the ligand, uridine-5'-monophosphate in grey.

**Figure 8:**
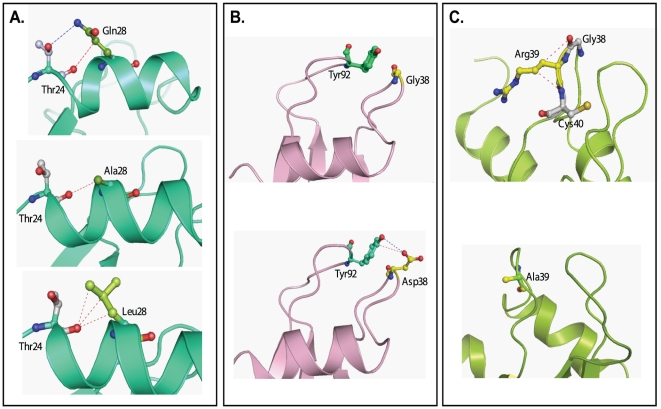
*In silico* analysis of HPR variants. The structures were drawn in PyMOL software using the coordinates of 1DZA (19). All important residues are shown in ball and stick model. The three panels show effect of respective mutations on various interactions in HPR variants. A. Gln28; B. Gly38; and C. Arg39. The hydrogen bond and van der Waal interactions are shown in blue and red dotted lines, respectively.

The superimposed structures of HPR and RNase A are very similar with a minimal root mean square deviation across main chain carbon atoms (Figure 7). The results of contact program revealed that main chain and side chain atoms of residues Gln28, Gly38 and Arg39 form 22, 14 and 19 interactions with protein atoms respectively (Table 5). Mutation of these residues with indicated residues, barring Gln28 to Leu, caused a significant change in the number of interactions mainly offered by side chain atoms (Table 5). As shown in Figure 8A, Gln28 and Thr24 form one hydrogen bond and one van der Waal interaction through their side chain atoms, and substitution of Gln28 with Ala results in the elimination of these interactions. However, when Gln28 is mutated to Leu, the strong interaction of Thr24 is maintained with Leu28 through the side chain atoms of respective residues (Figure 8A). In HPR, Gly38 lies in close vicinity of Tyr92, however there is no interaction between the two residues (Figure 8B). However, on mutating Gly38 to Asp two new interactions were observed between Asp38 and Tyr92 (Figure 8B). Arg39 does not show any interaction with any distant protein atom, though it shows few interactions with Gly38 and Cys40 due to its side chain atoms (Figure 8C). On mutating Arg39 to Ala these two interactions were altered (Figure 8C).

**Table 5:** Interactions of main chain and side chain atoms of Gln28, Gly38 and Arg39 with protein atoms.

Source atom	Target Residues	Distance (Å)	Source atom	Target Residues	Distance (Å)	Source atom	Target Residues	Distance (Å)
**Gln28**			**Leu28**			**Ala28**		
N	24 (THR)- O	2.97	N	24 (THR)- O	2.97	N	24 (THR)- O	2.97
	25 (TYR)- O	3.23		25 (TYR)- O	3.23		25 (TYR)- O	3.23
	26 (CYS)- C	3.17		26 (CYS)- C	3.17		26 (CYS)- C	3.17
	26 (CYS)- O	3.23		26 (CYS)- O	3.23		26 (CYS)- O	3.23
	27 (ASN)- C^β^	3.12		27 (ASN)- C^β^	3.12		27 (ASN)- C^β^	3.12
C^α^	27 (ASN)- O	2.75	C^α^	27 (ASN)- O	2.75	C^α^	27 (ASN)- O	2.75
C	29 (MET)- C	3.09	C	29 (MET)- C	3.09	C	29 (MET)- C	3.09
	27 (ASN)- C	3.08		27 (ASN)- C	3.08		27 (ASN)- C	3.08
	27 (ASN)- O	3.09		27 (ASN)- O	3.09		27 (ASN)- O	3.09
	30 (MET)- N	3.42		30 (MET)- N	3.42		30 (MET)- N	3.42
O	29 (MET)- C^α^	2.80	O	29 (MET)- C^α^	2.80	O	29 (MET)- C^α^	2.80
	29 (MET)- C	3.08		29 (MET)- C	3.08		29 (MET)- C	3.08
	32 (ARG)- C^β^	3.41		32 (ARG)- C^β^	3.41		32 (ARG)- C^β^	3.41
	32 (ARG)- C^τ^	3.25		32 (ARG)- C^τ^	3.25		32 (ARG)- C^τ^	3.25
	32 (ARG)- N	2.86		32 (ARG)- N	2.86		32 (ARG)- N	2.86
	31 (ARG)- N	3.26		31 (ARG)- N	3.26		31 (ARG)- N	3.26
	27 (ASN)- O	3.44		27 (ASN)- O	3.44		27 (ASN)- O	3.44
C^β^	29 (MET)- N	3.05	C^β^	29 (MET)- N	3.02	C^β^	29 (MET)- N	3.07
C^γ^	24 (THR)- O	3.33	C^γ^	24 (THR)- O	3.47			
N^ε2^	24 (THR)- O^γ1^	3.41	C^δ1^	24 (THR)- C	3.47			
**Gly38**			**Asp38**					
N	36 (THR)- C	3.39	N	36 (THR)- C	3.39			
	36 (THR)- O	3.06		36 (THR)- O	3.06			
	92 (TYR)- C^δ1^	3.48		92 (TYR)- C^δ1^	3.48			
	92 (TYR)- C^ε1^	3.43		92 (TYR)- C^ε1^	3.43			
C^α^	37 (GLN)- O	2.72	C^α^	37 (GLN)- O	2.72			
C	37 (GLN)- C	3.24	C	37 (GLN)- C	3.24			
	37 (GLN)- O	3.25		37 (GLN)- O	3.25			
	92 (TYR)- C^γ^	3.49		92 (TYR)- C^γ^	3.49			
	39 (ARG)- C^β^	3.35		39 (ARG)- C^β^	3.35			
O	92 (TYR)- C^γ^	3.49	O	92 (TYR)- C^γ^	3.49			
	39 (ARG)- C^α^	2.80		39 (ARG)- C^α^	2.80			
			C^β^	37 (GLN)- C	2.83			
				37 (GLN)- O	2.55			
				39 (ARG)- N	3.38			
			C^γ^	37 (GLN)- C	3.41			
				37 (GLN)- O	3.38			
			O^δ2^	37 (GLN)- O	3.41			
Arg39			Ala39					
N	37 (GLN)- C	3.16	N	37 (GLN)- C	3.16			
	37 (GLN)- O	3.17		37 (GLN)- O	3.17			
C^α^	38 (GLY)- O	2.80	C^α^	38 (GLY)- O	2.80			
C	40 (CYS)- C	3.16	C	40 (CYS)- C	3.16			
O	36 (THR)- C^α^	3.24	O	36 (THR)- C	3.36			
	36 (THR)- C	3.36		36 (THR)- C^α^	3.24			
	40 (CYS)- C	3.44		40 (CYS)- C	3.44			
	40 (CYS)- C^α^	2.82		40 (CYS)- C^α^	2.82			
C^β^	38 (GLY)- C	3.35	C^β^	38 (GLY)- C	3.24			
	40 (CYS)- N	3.30		38 (GLY)- O	3.42			
C^γ^	40 (CYS)- N	3.22		40 (CYS)- N	3.39			
C^δ^	111 (GLU)- O^ε2^	3.04						
C^ζ^	115 (TYR)- C^ε1^	3.38						
N^η1^	112 (GLY)- O	3.45						
	115 (TYR)- C^δ1^	3.30						
	115 (TYR)- C^ε1^	3.48						

## Discussion

HPR is a member of single strand preferring ribonuclease A superfamily. However, HPR exhibits a remarkably high activity against double stranded RNA. This high dsRNA cleavage activity of HPR suggests the enzyme to be playing a role in host defense. In this study we have investigated the role of glutamine 28, glycine 38 and arginine 39 in the dsRNA cleavage activity of HPR. RNase A has a series of base and phosphate binding sites, in addition to the catalytic site, that help in its binding to RNA. Evidence for specific binding extending beyond the active site has been obtained from crystallographic analyses of several RNase A-nucleotide complexes viz d(CpA) [18], ApC [19], pTp [20], and other studies including NMR [21], [22]. The crystal structure of a d(ApTpApApG).RNase A complex solved by X-ray diffraction shows the existence of a specific substrate recognition region on RNase A that extends beyond the active site [23]. According to this structure the side chains of Gln69, Asn71 and Glu111 may constitute a malleable binding site capable of establishing various hydrogen bonds depending on the nature of the stacked bases [23]. For polynucleotide substrates, remote subsites of interactions have been studied in detail in RNase A for poly (C) [24]–[26]. All these subsites are conserved in HPR, and the residues studied here are not part of those subsites. Figure 7 shows a superimposition of HPR and RNase A-Uridine-5′-monophosphate complex indicating that the substrate binds in pyrimidine binding site and the residues studied here are far away from the path of the substrate [27]. Our results show that the substitutions of residues Gln28, Gly38 and Arg39 alone or in combination in HPR do not affect its catalytic activity on single stranded RNA substrate poly (C) indicating that these residues in HPR are not involved in the interaction with long chain single stranded substrate poly(C). However, on dsRNA, poly(A).poly(U), HPR variants Q28A, R39A, Q28A/R39A and Q28A/G38D were 5- to 7-fold less active than HPR and this decrease was because of a parallel increase in their *K*_m_ values, as their *k*_cat_ were similar to that of the wild type HPR. In RNase A-polynucleotide catalysis, mutation of substrate binding subsite residues has resulted in 2–16-fold increase in the *K*_m_ of the variants [14], [15]. It appears that the side chains of Arg39 and Gln28 are involved in the interaction of HPR with double stranded substrate, and these interactions improve the catalysis of dsRNA. In RNase A, arginine 39 is one of the nine basic residues that are believed to form a multisite cationic region involved in protein-RNA interactions [28]. It is possible that arginine 39 improves dsRNA cleaving activity of HPR by helping the active site, which is located deep within the concave cleft of the enzyme, to access dsRNA. Clearly, other residues would be involved in converting this unproductive enzyme-dsRNA complex into productive ssRNA-enzyme complex. As compared to HPR, the R39A variant was found to be less efficient in melting dsRNA substrate analog poly(dA-dT).poly(dA-dT) indicating that arginine 39 could be contributing directly or indirectly towards the dsRNA melting activity of HPR. Although RNase A also contains arginine 39, it does not show dsDNA melting activity. It has been proposed that an asparatic acid present at position 38 in RNase A nullifies the positive charge of arginine 39 and prevents it from interacting with the negatively charged substrates [6]. Our *in silico* analysis confirms that though Arg39 side chain is not involved in holding the protein atoms together, it shows few interactions with Gly38 and Cys40, which are lost when Arg39 is substituted with Ala. It is shown earlier, that Gly38 in HPR has an important function, possibly, it may enhance the role of Arg39 as one of the many noncatalytic phosphate binding residues involved in the interaction of HPR protein with the double-helical substrate. In HPR, Gly38 is present on the surface in α2-β1 loop which forms a part of the V-shaped cleft in which the active site is located [16]. The absence of a larger side chain gives the polypeptide backbone at glycine residue much greater conformation flexibility than at other residues [16]. It appears that this conformational flexibility imparted to HPR around the active site, determines its ability to bind and melt duplex RNA, which is the first step in the hydrolysis of duplex RNA, and the presence of an aspartic acid instead of glycine at position 38, as in RNase A, diminishes the flexibility of the polypeptide backbone. In HPR, though Gly38 is in close vicinity of Tyr92, there is no contact between the two residues. However, mutation of Gly38 to Asp38 introduces one hydrogen bond and one van der Waal interaction with Tyr92.

The almost complete loss of dsRNA cleavage activity of R39A/G38D and Q28A/G38D/R39A can be explained by the absence of Arg39 which makes these proteins poor in melting the helix, and the presence of aspartic acid at position 38 which results in a compromised flexibility of the protein. The far-UV CD spectra and heat induced denaturation curves showed that R39A, Q28A and G38D had decreased stability. The effect of mutation on the stability of protein was more pronounced when R39A mutation was combined with Q28A, Q28L or G38D alone or Q28A and G38D together. The Q28L variant which was very similar to the native protein in terms of stability, showed similar DNA melting activity and in turn similar catalytic activity towards dsRNA as that of the native enzyme. Thus, stability also appears to be an important contributor in the DNA melting activity of HPR.

In BS-RNase, Leu28 is known to increase the propensity of domain swapping, and thus facilitating the formation of non-covalent dimer. However, analysis by native PAGE showed that Q28L mutation in HPR did not promote the process of dimerization (data not shown). Our study shows that a leucine at position 28 can substitute glutamine as Q28L variant showed similar stability, helix unwinding activity and dsRNA cleavage activity as that of HPR. The observation is further validated by *in silico* analysis that shows both Gln28 and Leu28 to have interaction with Thr24 which is lost in Q28A variant. The crystal structure of BS-RNase monomer, in which Leu28 occurs naturally, shows that Leu28, in spite of being a non polar residue remains partially exposed to the solvent, like Gln28 in HPR. Thus, leucine inspite of being non-polar may form similar interactions with dsRNA as glutamine.

In conclusion, we have studied the importance of two residues, arginine 39 and glutamine 28 for the functional activity of HPR and found that although they are not directly involved in catalysis, both arginine 39 and glutamine 28 could be facilitating the formation of a productive dsRNA-enzyme complex during dsRNA cleavage by HPR.

## Materials and Methods

### Materials

Single stranded RNA polymer, polycytidylic acid (potassium salt), poly(C) was from GE Healthcare. Double stranded homopolymer polyadenylic acid-polyuridylic acid sodium salt, poly(A).poly(U) and double stranded DNA poly(dA-dT).poly(dA-dT) were from Sigma. Other chemicals were of analytical grade.

### Construction and purification of HPR variants

The plasmid pHPR [29], which contains HPR cloned downstream of T7 promoter, was used as template for site-directed mutagenesis by overlapping primer extension by polymerase chain reaction. The mutations were confirmed by DNA sequencing. HPR and mutants were expressed in BL21 (λDE3) strain of *E. coli*. The recombinant proteins were found to accumulate in the inclusion bodies from where they were solubilized, refolded and purified as described earlier [30], [31]. Briefly, the inclusion bodies were solubilized in 6 M guanidine hydrochloride and renatured in 0.1 M Tris-HCl pH 8.0 containing 0.5 M arginine and 0.9 mM oxidized glutathione. The renatured proteins were dialyzed and purified by cation exchange chromatography using an S-sepharose column followed by gel filtration chromatography employing a Superose-12 column.

### CD spectral measurement of proteins

The far-UV CD spectra of HPR and its variants were recorded at 25°C in JASCO J-715 spectropolarimeter equipped with a Peltier-type temperature controller (PTC-348WI). The measurements were made in 50 mM phosphate buffer saline (PBS), pH 7.4 by using a quartz cell of 0.1 cm path length. Each spectrum was corrected for the blank contribution to the observed protein CD spectrum. The raw CD data at a given wavelength (λ) were converted into mean residue ellipticity [*θ*]_λ_, (deg cm^2^ dmol^−1^) by using the relation, [*θ*]_λ_ = *θ*_λ_ M_o_/10*lc*, where *θ*_λ_ is the observed ellipticity (millidegree) at wavelength λ, M_o_ is the mean residue weight of the protein, *c* is the protein concentration (mg/cm^3^), and *l* is the path length (cm). The concentration of HPR and its variants was calculated using *ε*_278_  = 7446 M^−1^cm^−1^.

### Steady state kinetics on poly(C)

The ribonucleolytic activity of HPR and variants was assayed on poly(C) as described earlier [10]. Briefly, an appropriate concentration of each protein that produced activity in the linear region at 20 min time point was used to determine the kinetic parameters of HPR and variants on single stranded RNA substrate poly (C). The enzyme concentration used was 70 pM and the substrate was varied from 16 to 640 µM. Different concentrations of substrate was incubated in 100 mM Tris-Cl (pH 7.5) with enzyme for 20 mins at 37°C. The reaction was stopped with 5% perchloric acid and 0.25% uranyl acetate. The undigested RNA was precipitated by incubating on ice for 30 min and separated from digested RNA by centrifugation. The amount of digested RNA was determined by measuring absorbance of the supernatant at 260 nm and the change in absorbance per unit time was converted to initial velocity. Kinetic parameters were deduced from the Michaelis-Menten plot of initial velocity vs substrate concentration. An absorption coefficient of 6200 M^−1^ cm^−1^ per nucleotide at 260 nm was taken for the calculation of substrate and product concentrations.

### Steady state kinetics on poly(A).poly(U)

For the determination of steady state kinetic parameters on double stranded RNA substrate, poly(A).poly(U), appropriate amount of the enzyme was mixed with different amounts of substrate in 0.1 M MOPS buffer (pH 7.5) containing 0.1 M sodium chloride [12]. The reaction mixture was transferred immediately to a cuvette of 1 cm path length, and the change in absorbance at 260 nm was monitored with time in a Perkin-Elmer Lambda Bio 20 spectrophotometer. Initial reaction velocity was calculated using Δ*ε*_260_ = 3400 M^−1^cm^−1^ for poly(A).poly(U). Substrate concentration was calculated using *ε*_260_ = 6500 M^−1^cm^−1^ for poly(A).poly(U). The substrate range used was 10 to 220 µM and the enzyme concentration used for HPR and variants was in the range 8.5–39.2 nM. The kinetic parameters were determined by non-linear regression analysis of the data fit to the Michaelis-Menten equation using Sigma plot software.

### Thermal denaturation of DNA substrate analog

Thermal denaturation of double stranded DNA poly (dA–dT).poly (dA–dT) in the absence and presence of proteins was monitored as described by Sorrentino et al. [5] with minor modifications. Briefly, 14 µg/ml DNA was mixed with 28 µg/ml of protein in 10 mM MOPS buffer containing 50 mM sodium chloride (pH 7.5) in a thermostatically controlled cuvette, and the change in absorbance at 260 nm was spectrophotometrically monitored in a temperature range of 35–80°C. The temperature was increased at 1°C per minute. The contribution of proteins to the absorbance of the mixture at 260 nm was negligible. The absorbance values of the free DNA and the complexes at various temperatures were normalized using the following equation:
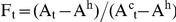



In the above equation subscript t represents temperature in °C at which measurements are made. F_t_ is the normalized absorbance indicating the fraction of melted DNA; A_t_ is the absorbance at the given temperature; A^h^ is the minimum absorbance over all the temperatures used and represents the absorbance solely due to helical duplexes; A^c^_t_ is the absorbance solely due to coil form of the polynucleotide. A^c^_t_ was obtained by fitting a least squares straight line among data points from 60 to 80°C.

### Thermal denaturation of proteins

Heat-induced denaturation of HPR and its variants was carried out in JASCO J-715 spectropolarimeter equipped with a Peltier-type temperature controller (PTC-348WI) with a heating rate of 1°C per minute. This scan rate was found to provide adequate time for equilibration. Changes in [*θ*]_222_ of each protein was measured in the temperature range 20–85°C. After denaturation, the sample was immediately cooled down to measure the reversibility of the reaction at different temperatures. It was observed that data from renaturation experiments fell on the denaturation curve. All solution blanks showed negligible change in ellipticity with temperature and were, therefore, neglected during the data analysis. Assuming that heat induces a transition two-state transition (N (native) state ↔ D (denatured) state), each denaturation curve was analyzed for the fraction denatured, *f*_D_ using the relation,
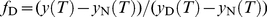
(1)where *y*(*T*) is the observed optical property at *T*°C, and *y*_N_(*T*) and *y*_D_(*T*) are the optical properties of the native and denatured protein molecules at *T*°C. *T*_m_, the midpoint of thermal denaturation, is the temperature (°C) at which *f*_D_ equals 0.5.

### In silico analysis of HPR variants

*In silico* site directed mutagenesis has been widely used to identify critical residue(s) for binding of a ligand/substrate or stability of protein. We have taken the coordinates of PDB ID 1DZA [16] for the structural analysis of HPR and its variants. Mutations in HPR were generated *in silico* using Swiss-Pdb Viewer Software [32]. The obtained computed structures of HPR variants were refined by energy minimization with CHARMM force field until the energy was reached [33]. The final predicted structures were evaluated sterically with PROCHEK [34]. The structures of HPR and its variants were drawn in PyMOL software [35] using the coordinates of both HPR and *in silico* mutated HPR variants. Contact distance of residues at 28, 38 and 39 position with that of other protein atoms was measured by the contact program from the CCP4 package [36].
