# Correction: Functional Role of Glutamine 28 and Arginine 39 in Double Stranded RNA Cleavage by Human Pancreatic Ribonuclease

**DOI:** 10.1371/annotation/68ded407-ac40-47b5-b3d7-966e116a28b2

**Published:** 2011-04-06

**Authors:** Md. Tabish Rehman, Punyatirtha Dey, Md. Imtaiyaz Hassan, Faizan Ahmad, Janendra K. Batra

The affiliations were missing information. The authors and correct affiliations are:"Md. Tabish Rehman2#, Punyatirtha Dey1#, Md. Imtaiyaz Hassan2, Faizan Ahmad2, Janendra K. Batra1*" "1 Immunochemistry Laboratory, National Institute of Immunology, Aruna Asaf Ali Marg, New Delhi 110067, India, 2 Centre for Interdisciplinary Research in Basic Sciences, Jamia Millia Islamia, New Delhi 110025, India"

